# Recombinant Interferon-γ Lentivirus Co-Infection Inhibits Adenovirus Replication *Ex Vivo*

**DOI:** 10.1371/journal.pone.0042455

**Published:** 2012-08-16

**Authors:** Ling Zhang, Sen Yin, Wanlong Tan, Dong Xiao, Yunceng Weng, Wenjing Wang, Tingting Li, Junwen Shi, Lifang Shuai, Hongwei Li, Jianhua Zhou, Jean-Pierre Allain, Chengyao Li

**Affiliations:** 1 Department of Transfusion Medicine, Southern Medical University, Guangzhou, China; 2 Department of Urology, Southern Medical University, Guangzhou, China; 3 Institute of Oncology, Southern Medical University, Guangzhou, China; 4 Guangzhou Military Centre of Disease Control, Guangzhou, China; 5 Laboratory of Lentiviruses and Horse Diseases, Harbin Veterinary Research Institute, Chinese Academy of Agricultural Sciences, Harbin, China; 6 Department of Hematology, University of Cambridge, Cambridge, United Kingdom; Shanghai Medical College, Fudan University, China

## Abstract

Recombinant interferon-γ (IFNγ) production in cultured lentivirus (LV) was explored for inhibition of target virus in cells co-infected with adenovirus type 5 (Ad5). The ability of three different promoters of CMV, EF1α and Ubiquitin initiating the enhanced green fluorescence protein (GFP) activities within lentiviruses was systematically assessed in various cell lines, which showed that certain cell lines selected the most favorable promoter driving a high level of transgenic expression. Recombinant IFNγ lentivirus carrying CMV promoter (LV-CMV-IFNγ) was generated to co-infect 293A cells with a viral surrogate of recombinant GFP Ad5 in parallel with LV-CMV-GFP control. The best morphologic conditions were observed from the two lentiviruses co-infected cells, while single adenovirus infected cells underwent clear pathologic changes. Viral load of adenoviruses from LV-CMV-IFNγ or LV-CMV-GFP co-infected cell cultures was significantly lower than that from adenovirus alone infected cells (*P* = 0.005–0.041), and the reduction of adenoviral load in the co-infected cells was 86% and 61%, respectively. Ad5 viral load from LV-CMV-IFNγ co-infected cells was significantly lower than that from LV-CMV-GFP co-infection (*P* = 0.032), which suggested that IFNγ rather than GFP could further enhance the inhibition of Ad5 replication in the recombinant lentivirus co-infected cells. The results suggest that LV-CMV-IFNγ co-infection could significantly inhibit the target virus replication and might be a potential approach for alternative therapy of severe viral diseases.

## Introduction

In recent years, recombinant lentivirus (LV) based on HIV-1 has uniquely progressed and become a focus of gene therapies for HIV diseases [1]–[4]. As a vector, lentivirus has the distinguishing property of being able to infect both dividing and non-dividing cells, and this ability has led to its development as a gene delivery vehicle [1], [5]. As the safest viral vector constructed by knocking out the virulent genes from HIV-1, the lentivirus encoding antiviral proteins might play a role in resistance to viral infection. A relevant study showed that lentivirus carrying interferon-β (IFNβ) gene could transduce CD4+/PBMC efficiently and highly express IFNβ, which significantly inhibited the replication of HIV-1 strain in CD4+/PBMC [6]. However, interferon-γ (IFNγ) has not been currently constructed within recombinant lentivirus for the purpose of antiviral therapy, particularly for DNA virus infection.

Here we hypothesized that efficient transduction of recombinant lentivirus expressing interferon gamma (IFNγ) in the host cells may play an important role as antiviral against the target virus through both lentivirus co-infection interference and IFNγ activity, which may provide an alternative pathway for controlling or curing severe viral diseases. This study extensively analyzed the ability of three different promoters within lentiviruses driving transgenic enhanced green fluorescence protein (GFP) expressing activities in various cell lines. The favorable promoter was used to construct the recombinant IFNγ lentivirus. Recombinant GFP adenovirus type 5 (Ad5) was used as a viral surrogate to examine the impact of lentivirus co-infection on target virus replication *ex vivo*.

## Results

### Generation of recombinant lentiviruses with three different promoters

**Figure 1:**
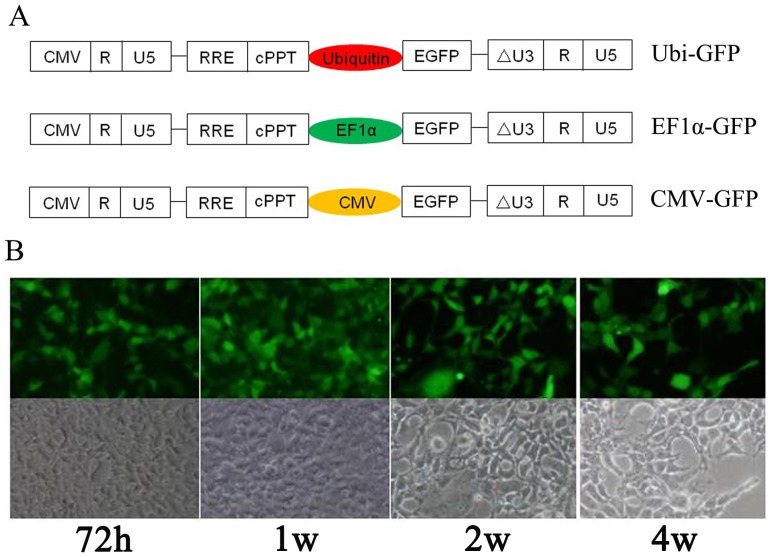
Characteristics of recombinant lentiviruses. (**A**) Schematic representation of lentiviral vector constructs. RRE: REV responsive element; cPPT: central polypurine tract; CMV: cytomegalovirus promoter; EF1α: human elongation factor 1 alpha promoter; EGFP: enhanced green fluorescence protein. (**B**) Morphology of passages from LV-CMV-GFP transduced 293A cells. The images were captured by a fluorescent or bright light microscope in 72 h after transduction or passaging over months.

The transfer expression plasmids pTY-EF1α-GFP, pTY-CMV-GFP and pTY-Ubi-GFP were constructed, which contained EF1α, CMV or Ubiquitin promoter, respectively. Those three lentiviral vectors were third generation of self-inactivating (SIN) vectors, in which the U3 region of 5′-LTR was replaced with cytomegalovirus (CMV) promoter. All three vectors contain a central polypurine tract (cPPT) that facilitate nuclear entry of the pre-integration complex (**Figure 1A**).

The lentiviruses (LV) carrying three different promoters were packaged in the transfer, shuttle and packaging plasmid DNA transfected 293T cells. Viral load of LV-CMV-GFP, LV-EF1α-GFP and LV-Ubi-GFP was quantified as containing 4.2×10^9^, 3.89×10^9^ or 5.37×10^9^ copies/ml from the supernatants of transfected cells, respectively. Six different cell lines were transduced by those three lentiviruses, in which LV-CMV-GFP transduced 293A cells were continually passaged keeping unchanged morphology and GFP stable over a one month period (**Figure 1B**).

### Transduction efficiency of lentiviruses with three promoters in various cell lines

**Figure 2:**
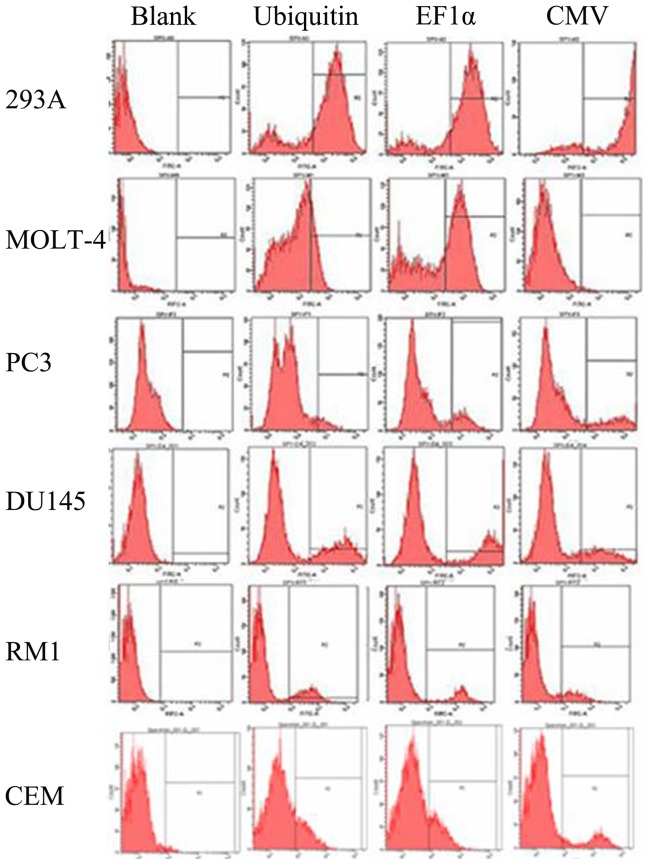
Detection of GFP expression driven by three different promoters within lentiviruses transduced various cells. GFP expressing cell counts and fluorescent signal intensity were measured by a flow cytometry in 72 h after lentivirus transduction.

The GFP activity under control of three promoters was observed by a fluorescent microscopy. The fluorescent signal intensities and efficiencies of lentiviruses transduced cells were measured by flow cytometry (**Figure 2**). In 293A and PC3 cells, CMV promoter initiated the highest activity of GFP expression. In contrast, CMV promoter showed much lower activity than the other two promoters in MOLT-4 and DU145 cells, while EF1α and Ubiquitin promoters were relatively stronger in those two cell lines. All three promoters were weaker in RM1 and CEM cells but EF1α had a relative higher activity for driving GFP expression. The transduction efficiency of those lentiviruses into various cell lines was ranging between 5% and 95% by flow cytometry (**Table 1**), which indicated that higher transduction was preferred by the lentivirus with a more efficient promoter in certain cell lines.

**Table 1:** Mean efficiency and fluorescent signal intensity of recombinant lentivirus transduced cells.

Cell lines	n	GFP expressing cell counts (%)		
		(Fluorescent signal intensity, ×10^4^)		
		LV-Ubi-GFP	LV-EF1α-GFP	LV-CMV-GFP
293A	3	89.13±4.28	79.90±6.25	94.83±2.87
		(8.85±0.44)	(5.67±1.66)	(22.56±0.78)
MOLT-4	3	64.83±13.01	74.27±2.14	5.87±1.70
		(0.61±0.04)	(2.82±0.68)	(0.33±0.15)
PC3	3	5.57±0.31	14.07±1.20	20.90±3.15
		(0.94±0.26)	(1.85±0.05)	(5.98±1.81)
DU145	3	24.10±5.53	25.13±4.95	16.87±3.92
		(3.47±1.72)	(6.98±3.83)	(1.87±0.03)
RM1	3	16.77±0.38	10.23±1.12	9.53±0.65
		(0.59±0.01)	(1.67±0.06)	(0.35±0.01)
CEM	3	15.70±0.56	8.80±0.06	4.90±0.38
		(0.30±0.00)	(2.00±0.01)	(0.32±0.01)

Mean GFP expressing cell count and fluorescent signal intensity were measured by a flow cytometry in 72 h after lentivirus transduction to cells.

### Production and characterization of recombinant IFNγ lentivirus

**Figure 3:**
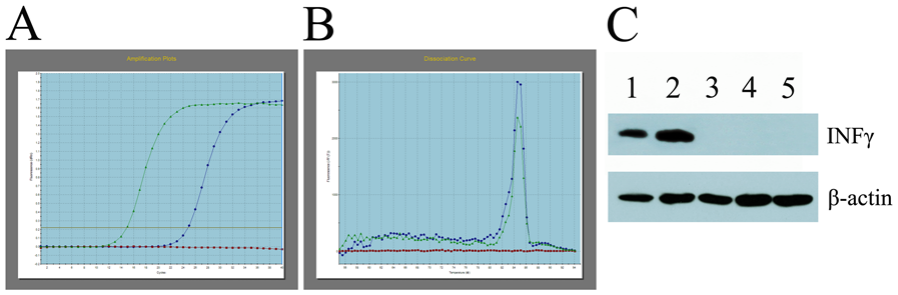
Infectivity of recombinant lentivirus to cells. (**A**) Amplification curve of IFNγ RNAs by SYBR-QPCR from lentiviruses transduced cells. The curves are representative of LV-CMV-IFNγ and IFNγ RNA positive control, respectively. (**B**) Dissociation curve (Derivative melting curve) of SYBR-QPCR was obtained from IFNγ RNA amplification. It is apparent that the point of infection (melting temperature of the amplicon) occurs at 85°C , and no contaminating product is present in this reaction. (**C**) Western Blot: lane 1, LV-CMV-IFNγ transduced cell lysate; lane 2, pTY-CMV-IFNγ plasmid transfected cell lysate; lane 3, LV-CMV-GFP transduced cell lysate control; lane 4, pTY-CMV-GFP plasmid transfected cell lysate control; lane 5, 293A cell lysate negative control. IFNγ was detected in 72 h after transduction or transfection to 293A cells.

Recombinant IFNγ lentivirus (LV-IFNγ) carrying CMV promoter (LV-CMV-IFNγ) was packaged in 293T cells. Viral load above 1×10^9^ copies/ml was observed in cell culture supernatant, and a viral load above 1×10^11^ copies/ml was obtained after concentration by filtration. Transcription and expression of IFNγ RNA were detected in LV-CMV-IFNγ transduced 293A cells by SYBR-QPCR and Western-blot (**Figure 3**), which indicated that LV-CMV-IFNγ was infectious. After 12 h transduction, high level of IFNγ secretion was detected (11.45 µg/ml and 10.44 µg/ml in supernatants of LV-CMV-IFNγ transduced 293A or CEM cells, respectively).

### Impact of recombinant lentivirus co-infection on replication of adenovirus in cell culture

**Figure 4:**
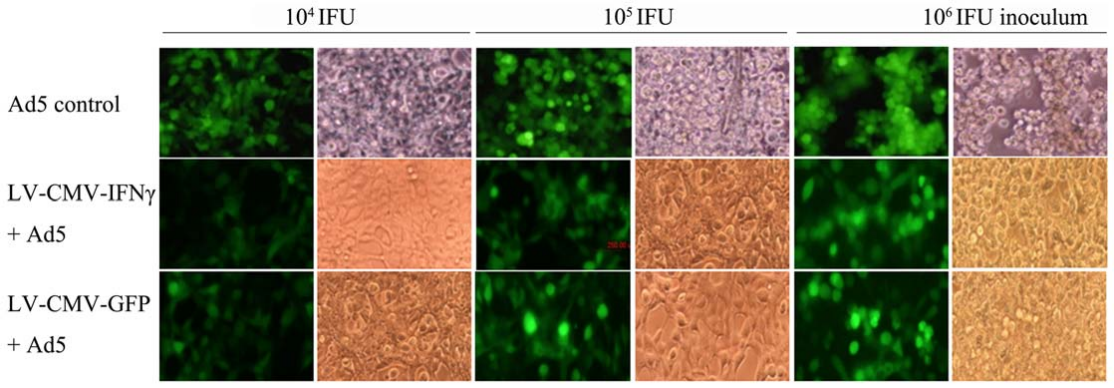
Observation of single adenovirus or lentivirus co-infected 293A cells. Ad5 control indicates the amount of 10^4^, 10^5^ and 10^6^ infectious units (IFU) of Ad5 alone infected 293A cells; LV-CMV-IFNγ**+**Ad5 and LV-CMV-GFP**+**Ad5 indicate the number of 10^8^ copies of LV-CMV-IFNγ or LV-CMV-GFP co-infected 293A cells with the amount of 10^4^, 10^5^ and 10^6^ IFU of Ad5, respectively. The images were captured by a fluorescent or bright light microscope in 72 h after single adenovirus infections or lentivirus co-infections.

**Figure 5:**
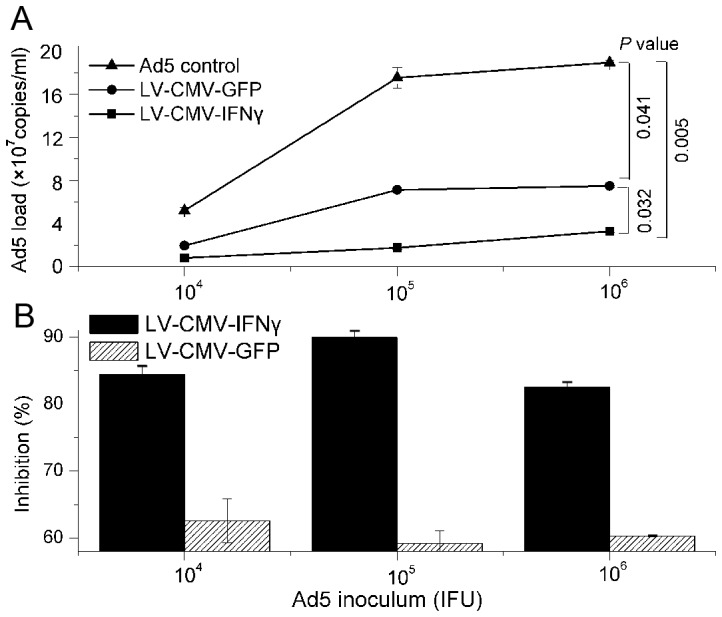
Comparison of Ad5 viral loads between single adenovirus infected and lentiviruses co-infected cell cultures. Ad5 control indicates that single adenovirus infected 293A cells, and LV-CMV-IFNγ and LV-CMV-GFP indicate that LV-CMV-IFNγ or LV-CMV-GFP co-infected 293A cells, respectively. (**A**) Ad5 viral loads were quantified by QPCR from single adenovirus infected 293A cell control or 10^8^ copies of each lentivirus co-infected cell cultures in three groups of 10^4^, 10^5^ and 10^6^ IFU of Ad5 inocula after 72 h cultivation, and data were obtained from three representative tests. The curves were plotted by mean ± SD of Ad5 viral loads. *P* values were calculated by two-way ANOVA test for comparing differences of Ad5 viral loads between single virus and lentivirus co-infected cell cultures or between lentivirus co-infections. (**B**) Inhibition (%) of Ad5 replication was calculated as viral load reduction from lentivirus co-infection to single Ad5 control in each inoculum group of cell cultures.

A day pre-seeded 1×10^5^ of 293A cells per well in the 24-well plate were individually inoculated with 10^4^, 10^5^ and 10^6^ infectious units (IFU) of recombinant GFP adenovirus type 5 (Ad5) and were co-infected with 10^8^ copies of LV-CMV-IFNγ or LV-CMV-GFP, respectively. The co-infected cells were observed and compared with a single adenovirus infection control, which showed the better morphologic condition, while adenovirus alone infected cells induced clear pathological changes (**Figure 4**). Viral load of adenovirus from LV-CMV-IFNγ or LV-CMV-GFP co-infected cell cultures was statistically lower than that from Ad5 alone infected cells (*P* = 0.005 and 0.041) (**Figure 5**), indicating that both recombinant lentiviruses could inhibit adenovirus replication in the co-infected cells. Between two lentivirus co-infections, Ad5 viral load from LV-CMV-IFNγ co-infected cell cultures was significantly lower than that from LV-CMV-GFP co-infected cells (*P* = 0.032) (**Figure 5A**), suggesting that interferon-γ rather than GFP could additionally enhance the inhibition of Ad5 replication in the co-infected cells. Replication of adenoviruses in 293A cell cultures was inhibited by those two lentivirus co-infections within a 59% to 90% range (**Figure 5B**). The largest reduction of Ad5 viral load was 83–90% (average 86%) from LV-CMV-IFNγ co-infected cell cultures, while 59–63% (average 61%) reduction of Ad5 viral loads was calculated from LV-CMV-GFP co-infected cells.

## Discussion

Up to now, with conventional vaccination ideas, scientists have experimentally and clinically attempted nearly every possible way of developing AIDS vaccines but all failed when tested in clinical trials [7]. However, recent epidemiological investigations showed that co-infection with a non-pathogenic flavivirus, GB virus C (GBV-C) replicating in human peripheral blood mononuclear cells (PBMCs) including CD4 positive T cells, could significantly benefit HIV-1 infected patients by delaying disease progression and prolonging HIV patient's survival [8]–[10]. Experimental data confirmed that GBV-C was able to inhibit HIV-1 replication *in vitro* [11], [12], and GBV-C viral load appeared to be inversely correlated with HIV-1 load in HIV-1 infected populations and to positively correlate with CD4 positive cell counts in symptomatic HIV-1 patients [13]. In the Sydney Blood Bank Cohort (SBBC) of long-term survivors, an attenuated nef-deleted HIV-1 strain was discovered and was ultimately found pathogenic in humans [14], [15]. The beneficial co-infection of HIV-1 infected patients with GBV-C or attenuated nef-deleted HIV-1 strain triggered exploring a different approach from the traditional vaccination for controlling HIV infection or disease progression. Substituting for vaccination, the persistent infection of CD4 positive cells with a constructed non-pathogenic virus in the HIV-infected hosts might competitively protect a sufficient number of target cells from being destroyed by HIV infection and, consequently, maintain a basic level of host immunity ensuring patient's survival.

The defective lentiviral vectors could interfere with replication of wild type HIV-1 in the transduced cells [1], [2]. Here we generated a recombinant interferon-γ lentivirus with self-inactivating replication derived from HIV-1 that essentially mimics an attenuated HIV-1 through viral interference and combines antiviral cytokine and gene therapy.

Highly effective expression of foreign transgenes within organs, tissues or cells is an important issue for lentiviral vector mediated transduction. It has been demonstrated that the higher transgenic expression can be obtained with optimization of transcriptional regulatory elements such as promoters [16]. In this study, three different promoters of CMV, EF-1α and Ubiquitin inserted within lentiviruses were compared for their ability to drive GFP expressions in transduced cell lines 293A, MOLT-4, DU145, PC3, CEM and RM1. The data collected indicated that one promoter appeared in various driven activities between different cell lines. Therefore, a proper promoter selection should be considered in order to achieve a high level of transgenic expression in the targetted host cell. The efficacy of CMV promoter for initiating GFP expression was found higher in 293A but lower in CEM cells. However, CMV promoter within lentivirus appeared to present high activity for driving IFNγ expression in both 293A and CEM cells, indicating that the favorable promoter might carry different ability for initiating different level of transgenic expression in certain cell lines.

Interferon gamma (IFNγ) is known as immune or type II interferon, which possesses antiviral activity directly affecting viral replication or modulating antiviral immune response to activate natural killer (NK) cells and cytotoxic T lymphocytes (CTL) by up-regulation of MHC class I and class II molecules in a wide variety of cells [17]–[22]. High viral load of recombinant IFNγ lentivirus was produced in this study. Secretion of IFNγ rapidly reached levels above 10 µg/ml in the supernatant of LV-CMV-IFNγ transduced 293A or CEM cell cultures, which was an effective concentration for antiviral activities *in vivo* [23].

Recombinant adenovirus type 5 carrying GFP (Ad5) was used as a viral surrogate for testing its interaction with lentiviruses in co-infected 293A cell cultures. The advantages of Ad5 to infect cells were rapid replication, accurate quantification, typical cytopathologic changes and observation by immunofluorescence, which made it an ideal target virus to evaluate the impact of recombinant lentivirus co-infection *ex vivo*. Recombinant lentiviruses expressing IFNγ or GFP under the control of a CMV promoter clearly inhibited adenovirus replication in co-infected cells (*P* = 0.005–0.041). In addition, high secretion of IFNγ (11.45 µg/ml) rather than GFP enhanced this inhibition through its antiviral activity (*P* = 0.032), for which 83–90% reduction of Ad5 viral load was obtained from LV-CMV-IFNγ co-infected cell cultures.

The mechanisms of recombinant lentivirus inhibiting HIV-1 replication were explored at least partly for TAR and RRE decoy effects of the vectors, competition for substrates necessary for the reverse transcription and RNA encapsidation, co-packaging or dimerization of HIV and vector RNAs at the dimer linkage structure (DLS) [24]–[27]. In this study, however, the inhibition of adenovirus replication in 293A cells by lentivirus co-infection could not be revealed by most of the above explanations, because adenovirus is a DNA virus and its replication mechanisms are different from RNA HIV-1. The study demonstrated that recombinant lentiviruses could inhibit both RNA and DNA virus replications in co-infected cells, suggesting that some universal mechanisms besides the antiviral activity of interferon were involved.

LV-CMV-IFNγ significantly inhibited the replication of adenovirus *ex vivo*, suggesting that the persistent and protective co-infection with recombinant interferon-γ lentivirus could be a potential approach for studying an alternative therapy of both DNA and RNA viral diseases, such as severe hepatitis B and C and HIV/AIDS. Prospective studies would examine the impact of recombinant IFNγ lentiviruses on replications of target viruses in host cells and animal models.

## Materials and Methods

### Gene and constructs/Ethics statement

Interferon gamma (IFNγ) gene was amplified and isolated from cDNA of Chinese volunteer peripheral blood cells in the laboratory. Written consent was obtained from all participants involved in the study and approved by Southern Medical University (SMU) Nan Fang Hospital Medical Ethics Committee (permit numbers: NFYY-2008-045) in accordance with national and institutional policies for medical ethics. Lentiviral transfer plasmids pTY-EF1α-IRES-GFP and pTY-Ubiquitin-GFP (pFUGW), packaging plasmids pMD2.G and psPAX2, and promoter plasmid pCMV-MCS were provided from the Department of Transfusion Medicine or the Institute of Oncology, Southern Medical University. The construct pTY-Ubi-GFP (Ubi-GFP) expresses the enhanced green fluorescence protein (GFP) under the control of Ubiquitin promoter [28]. The construct pTY-EF1α-GFP (EF1α-GFP) was generated by excising the IRES (internal ribosome entry site) fragment from pTY-EF1α-IRES-GFP at Mlu I and Sma I restriction sites. The plasmid pTY-CMV-GFP (CMV-GFP) was constructed from pTY-EF1α-GFP by replacing the EF1α promoter with human cytomegalovirus (CMV) promoter. Lentivirus transfer construct pTY-CMV-IFNγ was generated from above plasmid by replacing GFP with IFNγ.

### Cell lines

Cell lines 293T and 293A (human kidney epithelial cells), MOLT-4 (human acute lymphoblastic leukemia cell line), CEM (human lymphoblastic cell line), DU145 and PC3 (human prostate carcinoma cells) were provided from the laboratory and were initially purchased from Life Technologies™ (Invitrogen China Limited, Guangzhou, China). RM1 (murine prostate cancer cell) was provided by Wanlong Tan (Department of Urology, Southern Medical University, China), which was originally generated from C57BL/6 mice using the mouse prostate reconstitution model [29]. The cells were cultured in Dulbecco's modified Eagle's medium (DMEM) or RPMI-1640 supplemented with 10% fetal bovine serum (FBS), 100 units/ml penicillin, 100 µg/ml streptomycin, MEM Non-Essential Amino Acids Solution (10 mM), L-Glutamine (200 mM) in incubator with 5% CO_2_ and 37°C. Sera and media were purchased from a company (Invitrogen, Carlsbad, CA, USA).

### Recombinant lentivirus packaging

The lentiviral vectors used in this study are the third generation self-inactivating (SIN) vectors. A number of 3×10^6^ 293T cells were seeded in a T25 flask. On the following day, the culture medium was changed with 1.7 ml of Opti-MEM® I Reduced Serum Media. A mixture of 2.67 µg of shuttle plasmid pMD2.G, 5 µg of packaging plasmid psPAX2 and 6.67 µg of transfer expression plasmid DNA was prepared in lip2000 solution by using lipofectin transfection kit (Invitrogen, Carlsbad, CA, USA) and was co-transfected into 293T cells. The co-transfection solution was replaced by complete DMEM medium in 8 h post transfection. The packaged recombinant lentiviruses were harvested from the supernatant of cell cultures in 72 h after transfection. Lentivirus supernatants were treated by DNase I digestion. Viral load of recombinant lentivirus was quantified in copies/ml by real-time PCR with a standard curve generated from plasmid pTY-EF1α-GFP.

### Adenovirus

Recombinant adenovirus type 5 carrying GFP (Ad5) was packaged and produced in 293A cells as previously described [30]. The titer of infectious units (IFU) from adenovirus preparation was determined by using an Adeno-X Rapid Titer Kit (BD Biosciences, Palo Alto, CA, USA) according to the manufacturer's instruction. The stock contains 10^10^ IFU/ml of adenoviruses and stores at −80°C.

### Real-time PCR

Quantification of lentivirus or adenovirus was carried out by the real-time RT-PCR (RT-QPCR) or SYBR Green PCR (SYBR-QPCR). For detection of lentivirus, a region of 3′-LTR was selected to design the specific primers and probe as the followings [31]: Primer LV-F 5′-TAAAGCTTGCCTTGAGTGCT-3′, Primer LV-R 5′-GTCTGAGGGATCTCTAGTTACCAG-3′, LV-Probe 5′-(Hex) AGTAGTGTGTGCCCGTCTGTTGTGTG (BHQ2)-3′. For detection of adenovirus, a conserved region of the hexon gene was designed as the following primers [32]: Primer Ad-F 5′-GGTGGCCATTACCTTTGACTCTTC-3′ and Primer Ad-R 5′- CCACCTGTTGGTAGTCCTTGTATTTAGTATCATC -3′.

Lentivirus RNA or adenovirus DNA was prepared from cell cultures using the High Pure Viral Nucleic Acid Extraction kit (Roche Diagnostics GmbH, Mannheim, Germany). Two-step RT-QPCR or SYBR-QPCR was performed for quantification of lentivirus or adenovirus according to the protocols of manufacturer's instructions (Takara Biotechnology Co., Ltd, Dalian, China). The standard curve for lentivirus or adenovirus quantification was generated by serial dilutions of plasmid pTY-EF1α-GFP or pAdtrack DNA, respectively.

### Cell transduction *ex vivo*

A number of 1 to 2×10^5^ of cells of 293A, DU145, PC3, RM1, MOLT-4 or CEM were seeded in a well of 24-wells plate to obtain an appropriate density of 70–80% confluence on the following day. The cells in each well were transduced with 10^8^ particles of packaged recombinant lentiviruses in 2% FBS maintaining medium and incubated for 3 days at 37°C and 5% CO_2_. Gene expression of the transduced cells was examined by the immunoassays or observation of enhanced green fluorescent protein (GFP) on an Olympus Model BX41 fluorescent microscope (Olympus, Tokyo, Japan).

Efficiency of lentivirus-GFP transducing cells was analyzed by measurement of mean fluorescence signal in flow cytometry on a FACS Calibur (Becton Dickinson, MA, USA). Data were analyzed using CellQuest software (Becton Dickinson, MA, USA).

Expression level of IFNγ in supernatants or lysate from the transduced cell cultures was detected by Western-blot and commercially available enzyme-linked immunosorbent assay (ELISA) (R&D Systems, Inc. Minneapolis, MN, USA) according to the manufacturer's instruction.

### Statistical analysis

Experimental values are represented as the mean ±SD. SPSS13.0 software was used for data analysis. Two-way ANONA test was used to compare the difference of mean values of Ad5 viral loads between experimental and control groups. *p*<0.05 was statistically considered significant.

## References

[1] Klimatcheva E, Planelles V, Day SL, Fulreader F, Renda MJ, et al (2001) Defective lentiviral vectors are efficiently trafficked by HIV-1 and inhibit its replication. Mol Ther 3: 928–939. 11407907 10.1006/mthe.2001.0344

[2] Zeng L, Planelles V, Sui Z, Gartner S, Maggirwar SB, et al (2006) HIV-1-based defective lentiviral vectors efficiently transduce human monocytes-derived macrophages and suppress replication of wild-type HIV-1. J Gene Med 8: 18–28. 16142830 10.1002/jgm.825 PMC2825118

[3] Cao S, Wu C, Yang Y, Sniderhan LF, Maggirwar SB, et al (2011) Lentiviral vector-mediated stable expression of sTNFR-Fc in human macrophage and neuronal cells as a potential therapy for neuroAIDS. J Neuroinflammation 8: 48. 21569583 10.1186/1742-2094-8-48 PMC3118348

[4] Scherer LJ, Rossi JJ (2011) Ex vivo gene therapy for HIV-1 treatment. Hum Mol Genet 20 R1:R100–7. 21505069 10.1093/hmg/ddr160 PMC3095057

[5] Naldini L, Blömer U, Gallay P, Ory D, Mulligan R, et al (1996) In vivo gene delivery and stable transduction of nondividing cells by a lentiviral vector. Science 272: 263–267. 8602510 10.1126/science.272.5259.263

[6] Brule F, Khatissian E, Benani A, Bodeux A, Montagnier L, et al (2007) Inhibition of HIV replication: a powerful antiviral strategy by IFN-beta gene delivery in CD4+ cells. Biochem Pharmacol 74: 898–910. 17662695 10.1016/j.bcp.2007.06.036

[7] Kaiser J (2008) Review of vaccine failure prompts a return to basics. Science 320: 30–31. 18388263 10.1126/science.320.5872.30

[8] Xiang J, Wünschmann S, Diekema DJ, Klinzman D, Patrick KD, et al (2001) Effect of coinfection with GB virus C on survival among patients with HIV infection. N Engl J Med 345: 707–714. 11547739 10.1056/NEJMoa003364

[9] Tillmann HL, Heiken H, Knapik-Botor A, Heringlake S, Ockenga J, et al (2001) Infection with GB virus C and reduced mortality among HIV-infected patients. N Engl J Med 345: 715–724. 11547740 10.1056/NEJMoa010398

[10] Williams CF, Klinzman D, Yamashita TE, Xiang J, Polgreen PM, et al (2004) Persistent GB virus C infection and survival in HIV-infected men. N Engl J Med 350: 981–990. 14999110 10.1056/NEJMoa030107

[11] Nattermann J, Nischalke HD, Kupfer B, Rockstroh J, Hess L, et al (2003) Regulation of CC chemokine receptor 5 in hepatitis G virus infection. AIDS 17: 1457–1462. 12824783 10.1097/00002030-200307040-00006

[12] Xiang J, George SL, Wünschmann S, Chang Q, Klinzman D, et al (2004) Inhibition of HIV-1 replication by GB virus C infection through increases in RANTES, MIP-1alpha, MIP-1beta, and SDF-1. Lancet 363: 2040–2046. 15207954 10.1016/S0140-6736(04)16453-2

[13] Li C, Collini P, Danso K, Owusu-Ofori S, Dompreh A, et al (2006) GB virus C and HIV-1 RNA load in single virus and co-infected West African individuals. AIDS 20: 379–386. 16439871 10.1097/01.aids.0000200536.79360.03

[14] Deacon NJ, Tsykin A, Solomon A, Smith K, Ludford-Menting M, et al (1995) Genomic structure of an attenuated quasi species of HIV-1 from a blood transfusion donor and recipients. Science 270: 988–991. 7481804 10.1126/science.270.5238.988

[15] Gorry PR, Churchill M, Learmont J, Cherry C, Dyer WB, et al (2007) Replication-dependent pathogenicity of attenuated nef-deleted HIV-1 in vivo. J Acquir Immune Defic Syndr 46: 390–394. 17993857 10.1097/QAI.0b013e31815aba08

[16] Kim SY, Lee JH, Shin HS, Kang HJ, Kim YS (2002) The human elongation factor 1 alpha (EF-1 alpha) first intron highly enhances expression of foreign genes from the murine cytomegalovirus promoter. J Biotechnol 93: 183–187. 11738725 10.1016/s0168-1656(01)00388-1

[17] Pestka S, Langer JA, Zoon KC, Samuel CE (1987) Interferons and their actions. Ann Rev Biochem 56: 727–777. 2441659 10.1146/annurev.bi.56.070187.003455

[18] Giavedoni L, Ahmad S, Jones L, Yilma T (1997) Expression of gamma interferon by simian immunodeficiency virus increases attenuation and reduces postchallenge virus load in vaccinated rhesus macaques. J Virol 71: 866–872. 8995602 10.1128/jvi.71.2.866-872.1997 PMC191133

[19] Dale CJ, Zhao A, Jones SL, Boyle DB, Ramshaw IA, et al (2000) Induction of HIV-1-specific T-helper responses and type 1 cytokine secretion following therapeutic vaccination of macaques with a recombinant fowl poxvirus co-expressing interferon-gamma. J Med Primatol 29: 240–247. 11085586 10.1034/j.1600-0684.2000.290317.x

[20] Kim JJ, Yang JS, Manson KH, Weiner DB (2001) Modulation of antigen-specific cellular immune responses to DNA vaccination in rhesus macaques through the use of IL-2, IFN-gamma, or IL-4 gene adjuvants. Vaccine 19: 2496–2505. 11257383 10.1016/s0264-410x(00)00479-5

[21] Frese M, Schwärzle V, Barth K, Krieger N, Lohmann V, et al (2002) Interferon-gamma inhibits replication of subgenomic and genomic hepatitis C virus RNAs. Hepatology 35: 694–703. 11870386 10.1053/jhep.2002.31770

[22] Iida T, Kuwata T, Ui M, Suzuki H, Miura T, et al (2004) Augmentation of antigen-specific cytokine responses in the early phase of vaccination with a live-attenuated simian/human immunodeficiency chimeric virus expressing IFN-gamma. Arch Virol 149: 743–757. 15045561 10.1007/s00705-003-0229-z

[23] Younes HM, Amsden BG (2002) Interferon-gamma therapy: evaluation of routes of administration and delivery systems. J Pharm Sci 91: 2–17. 11782893 10.1002/jps.10007

[24] Hoglund S, Ohagen A, Goncalves J, Panganiban AT, Gabuzda D (1997) Ultrastructure of HIV-1 genomic RNA. Virology 233: 271–279. 9217051 10.1006/viro.1997.8585

[25] Corbeau P, Wong-Staal F (1998) Anti-HIV effects of HIV vectors. Virology 243: 268–274. 9568026 10.1006/viro.1998.9089

[26] An DS, Morizono K, Li QX, Mao SH, Lu S, et al (1999) An inducible HIV vector which effectively suppresses HIV replication. J Virol 73: 7671–7677. 10438857 10.1128/jvi.73.9.7671-7677.1999 PMC104294

[27] Bukovsky AA, Song JP, Naldini L (1999) Interaction of HIV-derived vectors with wild-type virus in transduced cells. J Virol 73: 7087–7092. 10400815 10.1128/jvi.73.8.7087-7092.1999 PMC112802

[28] Lois C, Hong EJ, Pease S, Brown EJ, Baltimore D (2002) Germline transmission and tissue-specific expression of transgenes delivered by lentiviral vectors. Science 295: 868–872. 11786607 10.1126/science.1067081

[29] Thompson TC, Southgate J, Kitchener G, Land H (1989) Multistage carcinogenesis induced by ras and myc oncogenes in a reconstituted organ. Cell 56: 917–930. 2538247 10.1016/0092-8674(89)90625-9

[30] Li HW, Gao YX, Raizada MK, Sumners C (2005) Intronic enhancement of angiotensin II type 2 receptor transgene expression in vitro and in vivo. Biochem Biophys Res Commun 336: 29–35. 16122703 10.1016/j.bbrc.2005.08.035

[31] Fischetti L, Opare-Sem O, Candotti D, Lee H, Allain JP (2004) Higher viral load may explain the dominance of CRF02_AG in the molecular epidemiology of HIV in Ghana. AIDS 18: 1208–1210. 15166539 10.1097/00002030-200405210-00017

[32] Garnett CT, Erdman D, Xu W, Gooding LR (2002) Prevalence and quantitation of species C adenovirus DNA in human mucosal lymphocytes. J Virol 76: 10608–10616. 12368303 10.1128/JVI.76.21.10608-10616.2002 PMC136639

